# Neuromyelitis Optica Spectrum Disorder Attack Triggered by Herpes Zoster Infection

**DOI:** 10.1155/2020/6151258

**Published:** 2020-04-25

**Authors:** Emanuela Claudia Turco, Erica Curti, Valentina Maffini, Francesco Pisani, Franco Granella

**Affiliations:** ^1^Child Neuropsychiatric Unit, Maternal and Child Health Department, Parma University Hospital, Via Gramsci 14, 43126 Parma, Italy; ^2^Unit of Neurosciences, Department of Medicine and Surgery, University of Parma, Italy; ^3^Emergency and General Pediatric Unit, Maternal and Child Health Department, University Hospital of Parma, Italy; ^4^Unit of Neurology, Department of Medicine, Parma University Hospital, Italy

## Abstract

Neuromyelitis optica spectrum disorder is a severe autoimmune disease of the central nervous system characterized by recurrent inflammatory events primarily involving the optic nerves and spinal cord. Recently, a triggering role of infectious events in the development of NMOSD has been suggested. Varicella zoster virus is the most common viral infection involved, though the linkage with anti-aquaporin-4 antibodies is so far unknown. We report, to the best of our knowledge, the first pediatric case report about NMOSD relapse triggered by herpes zoster infection. The strict temporal relationship between VZV infection and NMO attacks seems to be more than simply due to chance; however, further reports are needed to be confirmed.

## 1. Introduction

Neuromyelitis optica spectrum disorder (NMOSD), previously known as Devic's disease, is an immunomediated inflammatory disease of the central nervous system (CNS), predominantly affecting the optic nerves and spinal cord (SC). A pathogenic role has been attributed to specific autoantibodies that target aquaporin-4 (AP4) channels, playing an important role in brain water homeostasis [1]. The pathogenic mechanisms of NMOSD have been sufficiently elucidated, yet the etiology is poorly understood. Some studies have suggested a triggering role for infectious agents, particularly varicella zoster virus (VZV) [2]. Here, we report a case of NMOSD attack in a 17-year-old patient occurring after reactivation of VZV. To the best of our knowledge, this is the first pediatric report in which herpes zoster infection preceded a clinical attack of NMOSD.

## 2. Case Report

A previously healthy immunocompetent 17-year-old female was admitted to our hospital in August 2018 for sensory impairment, pain in her right arm, and transient blurred vision.

About three weeks before, she had had a second vesicular rash on her right armpit and chest (T2), for which she underwent a 10-day antiviral treatment with oral acyclovir (800 mg bid).

Vital signs were normal. At the neurological examination, we observed right eye mydriasis, piloerection, poikilothermia, mild hypoesthesia, and pain in the right arm and trunk in the T2-T3 dermatomes.

Her medical history reported hospitalisation six months earlier due to sudden onset of incoercible vomiting and fever of unknown etiology. Clinical examination, laboratory tests, and multiple investigations including brain CT scan and esophagogastroduodenoscopy revealed no abnormal findings. This episode was followed by the first herpes zoster eruption, involving right T2 dermatome, successfully treated with oral acyclovir.

**Figure 1:**
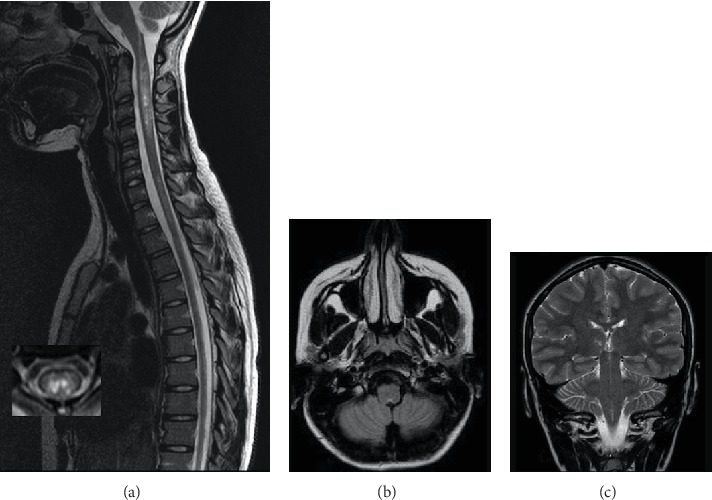
(a) Sagittal and axial T2-weighted spinal MR imaging showing hyperintense lesions longitudinally extended from C1 to C5 and from C6 to T6, involving the central spinal cord, with bright spotty lesions. (b) Axial T2-weighted fluid-attenuated inversion recovery (FLAIR) with hyperintense lesion in the brainstem involving the dorsal medulla (area postrema). (c) Coronal T1-weighted brain MRI shows a lesion on the right ventrothalamic area.

On admission in August, MRI scan showed multiple T2 hyperintense lesions in both the brain and the SC. Lesions involved the area postrema, right ventrothalamic area, periaqueductal gray, optic tracts, and cervical and thoracic regions, longitudinally extended from C1 to C5 and from C6 to T6 and axially involving two-thirds of the SC. The cervical SC showed swelling and T2 very hyperintense lesions, so-called bright spotty lesions, and nodular and meningeal gadolinium enhancement on T1-weighted sequences (see Figure 1).

Routine blood tests, including blood cell count, coagulation, and thyroid, hepatic, and renal function studies, were normal. Serum autoantibody screening was positive for AQP4 antibody, negative for myelin oligodendrocyte glycoprotein antibody, and mildly positive for antinuclear antibodies and myelin-associated glycoprotein antibodies. Serology for neurotropic infectious agents showed no significant remarks except for VZV IgM and IgG positivity. Cerebrospinal fluid (CSF) revealed mild lymphocytic pleocytosis (32 cell/mm^3^), increased total protein (74 mg/dL), and two oligoclonal bands, both in the CSF and blood (mirror pattern). PCR test for VZV DNA in the CSF was negative, while IgM VZV-specific antibody index was high (7.10; reference range 0.3–2.0), suggesting intrathecal synthesis.

A diagnosis of AQP4 NMOSD was made based on clinical symptoms (intractable vomiting suggesting an area postrema syndrome and longitudinally extensive transverse myelitis (LETM)) and laboratory and neuroimaging findings. The patient was administered a high dose of intravenous methylprednisolone (1 g/day for five days) and intravenous acyclovir (500 mg tid for 11 days), leading to resolution of symptoms. She continued oral antiviral treatment after being discharged (acyclovir 800 mg tid).

Rituximab was started as a disease-modifying treatment at a dose of 1.000 mg twice two weeks apart.

At 6-month MRI control, cervical lesions were markedly decreased, although gadolinium enhancement persisted at the bright spotty lesion sites. Right ventrothalamic area showed mild T2 hyperintensity.

## 3. Discussion

NMOSD is a rare inflammatory demyelinating disease of the CNS that predominantly targets optic nerves and SC, resulting in optic neuritis (ON) and transverse myelitis extending over 3 or more vertebral segments with contrast enhancement that may be persistent at follow-up [3]. Other clinical features include area postrema syndrome (intractable hiccups, nausea/vomiting) and brainstem and diencephalic syndromes such as narcolepsy/hypersomnolence and endocrine dysfunction [1]. Pediatric-onset NMOSD accounts for 3–5% of all NMOSD cases, depending on the diagnostic criteria applied and the inclusion of AQP4 antibody testing. In a large case series collected at Mayo Clinic, 5% of AQP4 antibody-positive patients were children (<18 years), with a mean age at clinical onset of 12 years, and clear female preponderance (7 : 1). Female preponderance in children is less overwhelming in comparison to adults (F : M ratio of 3 : 1 compared to 9 : 1 in adults), and the disease is more often monophasic [4]. When NMOSD is multiphasic, the relapses mostly occur within one year from onset [1]. The attacks are frequently severe, and recovery is poor. Consequently, neurological impairment accumulates and prognosis is poorer if compared to childhood multiple sclerosis [4]. A triggering role of infectious events in the development of NMOSD has recently been suggested [5]. Viral infections precede NMOSD in 15–35% of the cases, but their linkage with anti-AP4 antibodies is as yet unknown [2]. Among viral infections preceding NMOSD, the most common are VZV [6–8], mumps [9], HIV [10], and Epstein–Barr virus [11]. Possible mechanisms to explain the association of autoimmunity with virus infection are bystander activation, in which microbes damage AP4-rich tissue provoking the activation of AP4-specific T- and B-cells against the CNS or molecular mimicry, in which the activation of B-cells produces antibodies that recognize both microbial epitopes and self-epitopes [2]. Recent data have suggested that infection could activate an inflammatory cascade documented by an increase in CSF IL-6, which promotes anti-AP4 antibody production from plasmablasts. In our case, one of the abovementioned mechanisms could explain NMOSD relapse triggered by zoster, confirmed by high-IgM VZV-specific antibody index, a marker of CNS synthesis which is more reliable than the presence of VZV DNA [12]. On the other side, NMOSD could have altered cell-mediated immunity with an increased incidence of zoster outbreaks. In fact, repeated zoster eruptions in immunocompetent youngsters are very uncommon [13]. To the best of our knowledge, this is the first pediatric case report about NMOSD relapse potentially triggered by herpes zoster infection. The strict temporal relationship between VZV infection and NMO attacks seems to be more than simply due to chance; however, further reports are needed to be confirmed.

## Data Availability

No data were used to support this study.
